# Web-Based System to Capture Consistent and Complete Real-world Data of Physical Therapy Interventions Following Total Knee Replacement: Design and Evaluation Study

**DOI:** 10.2196/37714

**Published:** 2022-10-27

**Authors:** Patricia D Franklin, Carol A Oatis, Hua Zheng, Marie D Westby, Wilfred Peter, Jeremie Laraque-Two Elk, Joseph Rizk, Ellen Benbow, Wenjun Li

**Affiliations:** 1 Department of Medical Social Sciences Feinberg School of Medicine Northwestern University Chicago, IL United States; 2 Arcadia University Glenside, PA United States; 3 Department of Orthopedics and Physical Rehabilitation University of Massachusetts Medical School Worcester, MA United States; 4 Centre for Hip Health and Mobility Vancouver Coastal Health Research Institute Vancouver, BC Canada; 5 Department of Orthopedics, Rehabilitation, and Physical Therapy Leiden University Medical Center Leiden Netherlands; 6 HealthPRO Heritage Bellevue, WA United States; 7 Cascade Rehabilitation Associates Everett, WA United States; 8 Magee Rehabilitation Hospital Philadelphia, PA United States; 9 Department of Public Health University of Massachusetts-Lowell Lowell, MA United States

**Keywords:** structured data, web-based clinical data capture, physical therapy, total knee replacement, electronic health records, real-world evidence, real-world data, data, therapy, knee, knee replacement, clinical intervention

## Abstract

**Background:**

Electronic health records (EHRs) have the potential to facilitate consistent clinical data capture to support excellence in patient care, quality improvement, and knowledge generation. Despite widespread EHR use, the vision to transform health care system and its data to a "learning health care system" generating knowledge from real-world data is limited by the lack of consistent, structured clinical data.

**Objective:**

The purpose of this paper was to demonstrate the design of a web-based structured clinical intervention data capture system and its evaluation in practice. The use case was ambulatory physical therapy (PT) treatment after total knee replacement (TKR), one of the most common and costly procedures today.

**Methods:**

To identify the PT intervention type and intensity (or dose) used to treat patients with knee arthritis following TKR, an iterative user-centered design process refined an initial list of PT interventions generated during preliminary chart reviews. Input from practicing physical therapists and national and international experts refined and categorized the interventions. Next, a web-based, hierarchical structured system for intervention and intensity documentation was designed and deployed.

**Results:**

The PT documentation system was implemented by 114 physical therapists agreeing to record all interventions at patient visits. Data for 161 patients with 2615 PT visits were entered by 83 physical therapists. No technical problems with data entry were reported, and data entry required less than 2 minutes per visit. A total of 42 (2%) interventions could not be categorized and were recorded using free text.

**Conclusions:**

The use of user-centered design principles provides a road map for developing clinically feasible data capture systems that employ structured collection of uniform data for use by multiple practitioners across institutions to complement and augment existing EHRs. Secondarily, these data can be analyzed to define best practices and disseminate knowledge to practice.

## Introduction

The health care system in the United States has moved aggressively in the last decade to the use of electronic health records (EHRs). A primary goal driving the transition to an EHR is the EHR’s potential to facilitate consistent data capture to support patient care and quality improvement in health care [1,2]. Moreover, routine collection of clinical data, in conjunction with insurance claims data, has the potential to enhance comparative effectiveness research (CER), all leading to improved patient outcomes. "Pragmatic trials" using real-world evidence from EHRs can include many more diverse individuals recruited from real-world settings and can assess the interventions provided during standard clinical care. In contrast, the usefulness of traditional randomized controlled trials has been limited by the relatively small study sample sizes, the tightly controlled inclusion and exclusion criteria of participants, and the tightly regimented interventions tested [3,4]. The realization of this vision will transform the clinical care system to a “learning health care system” to generate new knowledge from real-world data, while providing optimal care to today’s patients. In contrast to this vision, data regarding the EHR’s ability to improve clinical care, clinical research, and ultimately patient outcomes are limited. Bartlett et al [5] report that only 15% of US-based clinical trials published in 2017 in high-impact journals could be replicated using data found in EHRs or claims data. The investigators note that fewer than 40% of the reported interventions studied in randomized controlled trials could be assessed using EHR data. The authors suggest that improved EHR systems with predefined, consistent data capture might enhance the ability to study interventions via EHRs.

As an example, osteoarthritis (OA) is the most common disabling condition in the United States, and knee OA is among the most prevalent OAs [6,7]. When knee OA symptoms persist despite medical care and physical therapy (PT), total knee replacement (TKR) surgery is commonly elected. However, limited CER evidence exists to define the components of optimal post-TKR PT to achieve peak knee performance and physical function. A 2018 retrospective study [8] of PT paper records from patients seen at home or in ambulatory settings found that of 156 records available for review, only 112 provided sufficient intervention details to assess the quality of even a portion of the interventions. Review of those records revealed that interventions varied widely among physical therapists, with only 5 exercises reported in more than 50% of the records. Review also suggested that dosage of strengthening exercises might be inadequate to derive a physiological response. However, documentation was limited by incompleteness, illegibility, lack of consistent vocabulary, and the use of jargon.

Beyond incomplete and inconsistent EHR documentation, generalizable research requires integrating data across locations and time. Today’s EHRs vary in structure, functions, and their ability to capture structured data and extract and integrate existing data. Inconsistent discrete variable definitions, broad use of free-text fields, and limited embedded technical functions to identify post-TKR patients and extract data are barriers [2]. Today’s PT practices use EHRs to serve billing and administrative functions, but nonstructured treatment notes persist and perpetuate the challenge of using real-world data to define optimal PT practice in patients post TKR.

The purpose of this paper is to demonstrate the ability of a user-designed, web-based data capture system to track detailed, complete, and quantifiable PT interventions in patients following TKR to serve CER. This paper presents the development, deployment, and assessment of a structured data capture system for physical therapists treating patients in any ambulatory setting following TKR. The future goal of this data capture system is to describe and quantify the interventions provided by physical therapists to patients in all ambulatory care settings after TKR, in preparation for a pragmatic study to determine the PT interventions associated with optimal functional outcomes. Although the data capture system presented in this paper is designed for the specific patient population with knee OA post TKR surgery, albeit one that constitutes a large proportion of ambulatory PT care, we believe the existing system can be applied to many patient populations with minor modifications. More importantly, this paper offers design principles and a road map for developing clinically feasible web-based data capture systems that employ a structured collection of uniform clinical data, allowing use by multiple practitioners across institutions to complement and augment existing EHRs.

## Methods

### Ethics Approval

This research was approved by the Human Subjects Review Board at the University of Massachusetts Chan Medical School (H00012294_19).

### Patient Involvement

Development of an interoperable data capture system involved the following two distinct tasks proceeding in parallel: (1) identification of the relevant content to be captured for a thorough description of the PT intervention and (2) construction of a user-friendly, Health Insurance Portability and Accountability Act (HIPAA) Privacy Rule–compliant web-based data capture method for use across diverse PT practices. The following section presents these two tasks separately.

### Content Development

To identify the PT intervention content to be captured, we used an iterative user-centered design process building on the initial list of treatments generated during our retrospective chart review. The original list of interventions identified by chart review was entered into a Microsoft Excel spreadsheet and provided to 7 physical therapists and PT interns at 4 ambulatory PT clinics. The clinics were located in geographic regions that were different from those of clinics used in the original study. The 7 physical therapists and interns were asked to record their interventions on the spreadsheet and to add any interventions they used that were not listed on the spreadsheet. The original list of interventions was revised using the comments from the new set of users.

The revised list was then sent to 4 nationally recognized experts in PT post TKR care. These experts were asked to review the list of interventions and revise them as needed. All 4 experts shared the list with at least one practicing clinician for additional input. We interviewed all 4 national experts to review their comments and suggestions. The suggestions were compiled in the next version of the intervention list and returned to the national experts for further comment. Another round of revisions occurred. These revisions included dividing the list of interventions into 8 distinct categories. Finally, the next version of the intervention list was sent to 2 international physical therapist experts. They reviewed the list of interventions and discussed their suggestions in a series of 2 conference calls with the investigators. These discussions included suggestions on how to describe intensity and dosage. The investigators incorporated the suggestions and developed a final menu of possible interventions that could be provided by a physical therapist in an ambulatory setting to patients post TKR.

The process of review and revision took approximately one year to complete and resulted in a list of 141 interventions organized in 8 categories. Each intervention included additional parameters used to describe dosage and intensity. A file of intervention definitions was also generated, so clinicians could recognize an intervention by the definition, regardless of the name of the intervention (Multimedia Appendix 1).

### Informatics Development

**Figure 1:**
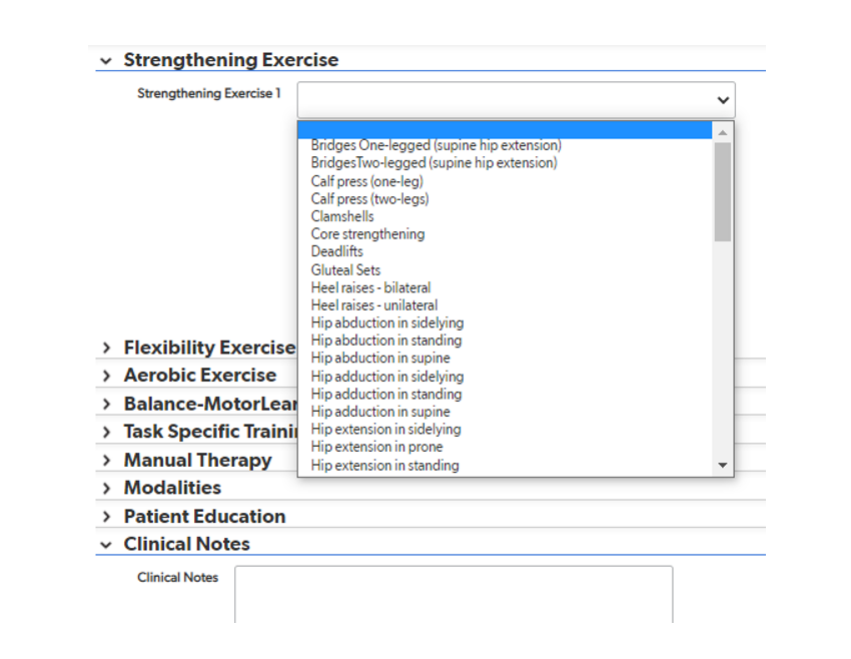
Sample structure of web-based physical therapy intervention capture system.

The primary purpose of this research was to validate and refine, as needed, the PT intervention documentation system prior to future integration with EHRs. Because this documentation would supplement the existing PT EHR system, efficient documentation and ease of use were priorities. The informatics team identified a HIPAA-compliant web-based software (Quickbase) that can capture discrete PT interventions and intensity details. Priority features included (1) secure and simple log-in and patient registration for PT efficiency and (2) hierarchical documentation structure to allow the physical therapist to quickly review the 8 categories and select interventions within only the relevant categories for each PT visit (Figure 1).

### Log-in and Patient Registration

Each physical therapist is assigned a unique log-in account and the password is updated every 60 days for data security purpose. The home page of the system includes an “Add New Patient” button from which the physical therapist enters basic patient information to register a new patient. The system automatically generates a unique ID for each patient.

### Intervention: Hierarchical Documentation of Interventions and Intensity

**Figure 2:**
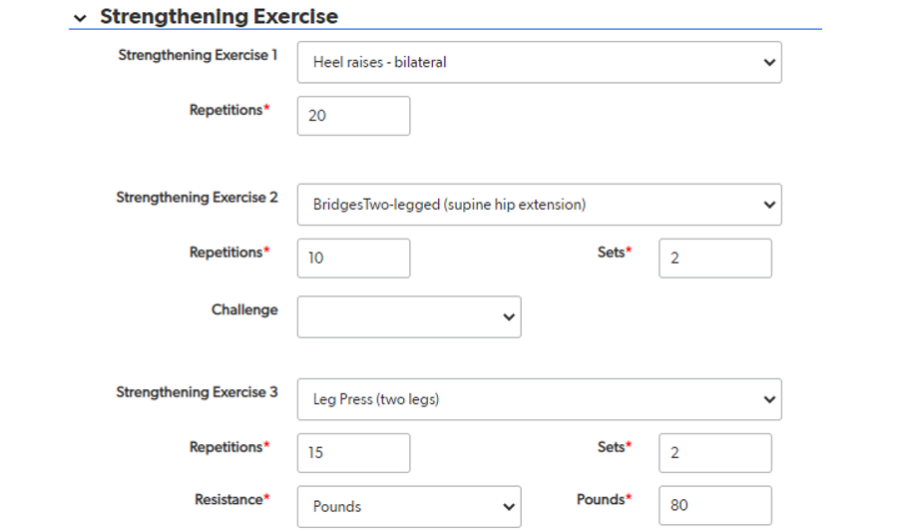
Data entry fields for physical therapy intervention intensity details, including repetitions, sets, resistance, and other related options.

Once a patient is registered in the system, an “Add Visit” button is displayed on the patient record. The physical therapist can add as many PT visits as needed for one patient, and each visit is assigned a record ID as well. On each visit page, the visit date and the interventions provided by the physical therapist are entered. The intervention data collection is structured by category. For each of the 8 categories, the physical therapist selects interventions from the list within the relevant category. Once the intervention is selected, repetitions, sets, resistance, and other related fields appear for data entry (Figure 2). A complex branching logic was built to support the entry screen that displays or hides the data fields for each selected intervention. This hierarchical structure provides an efficient and organized user interface for detailed and accurate intervention documentation.

**Figure 3:**
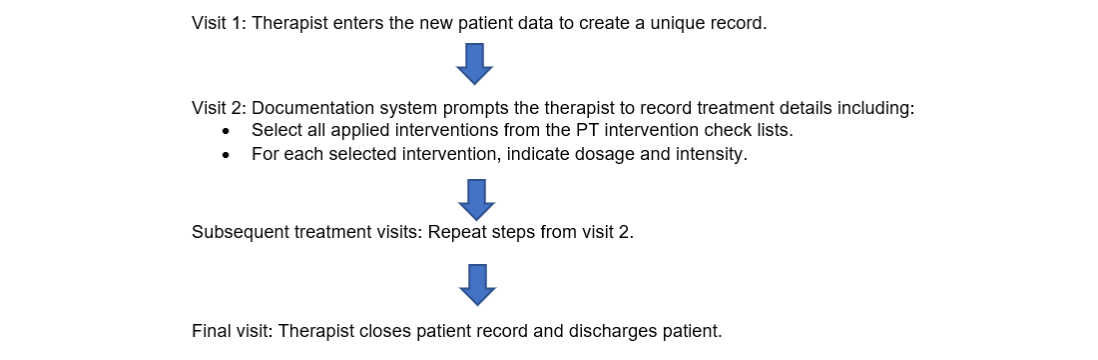
Workflow for physical therapist entering physical therapy intervention documentation.

The workflow for the physical therapist entering documentation data into the system is listed in Figure 3. This process exactly parallels how the EHR fits into the PT clinical workflow.

### System Deployment

We deployed the data capture system in 8 PT practices with 33 different office sites; 3 practices were located in central Massachusetts, 1 in Rhode Island, and 4 in southeastern Pennsylvania. A total of 107 physical therapists and 7 physical therapist assistants who treat patients post TKR at these practices agreed to document in the intervention system at each patient visit. Each physical therapist agreed to help identify eligible patients, complete a brief survey describing his or her educational and professional background, and enter the complete intervention data for every visit for up to 5 enrolled patients. Participating practices were reimbursed US $50 for each completed patient record documenting the content of each PT visit. Each participating physical therapist attended one or two 45-minute web-based training sessions to learn about the study and to learn how to use the data capture system.

### Assessment

The clinical feasibility of the data capture system was assessed in multiple ways. Therapists could contact IT support if they experienced problems with the website or technical difficulties in entering data. System function was assessed by the number and type of IT support contacts during the study. Physical therapists were also instructed to use a “Clinical Notes” text box in the data capture system to identify any interventions they used but were unable to find in the intervention menus. Therapists could also use the text box to add additional information that they wished to include for daily documentation. At least two trained PT reviewers reviewed the “Clinical Notes” text boxes to determine if they contained (1) additional information about interventions already included in the menus, (2) interventions that were available in the menu but not entered, or (3) interventions not represented in the menus. To evaluate the completeness of the documentation system, we identified the frequency of visits in which the text box was used, the frequency of visits where interventions were identified in the clinical texts but not entered in the menus, and the number of physical therapists associated with these texts. We also determined how often interventions listed in the text box were unavailable in the menus.

### Statistical Methods

We used descriptive analysis of aggregate data on the use of the PT data reporting system. No other statistical analyses were used.

## Results

### Content and Informatics Development

The final post-TKR PT intervention system is a web-based, menu-driven data collection system using a HIPAA-compliant platform. Interventions are organized into 8 categories, each with its own drop-down menu. The categories include the following: strengthening exercises; flexibility exercises; aerobic exercises; balance, mobility, and agility; task-specific training; manual therapy; modalities; and patient education. The number of possible interventions varied within each of these categories from a high of 62 possible knee or hip strengthening interventions to a low of 6 possible patient education interventions. Within each intervention, additional drop-down menus appear to describe dose and intensity of each intervention.

The original menus included 141 interventions; however, after monitoring the “Clinical Notes” text boxes for approximately two months, two additional interventions (n=143) were added to the menus, and definitions for 3 interventions were revised for clarity.

### System Deployment

A total of 107 therapists and 7 physical therapist assistants were trained in data entry. Over a period of approximately two years, data for 161 patients with 2615 patient visits were entered by 83 physical therapists or physical therapist assistants. Only therapists and assistants treating participating patients with new unilateral TKRs during the study recruitment period entered data. The characteristics of the participating patients are reported in Table 1 and are consistent with the national averages of patients receiving TKR.

**Table 1:** Patient characteristics (N=161).

Characteristics	Values
Age (years), mean (SD)	66 (8.4)
Female, n (%)	108 (67)
BMI, mean (SD)	30.4 (5.4)
Visits, mean (SD)	15.8 (9.8)
Side of surgery (right), n (%)^a^	42 (26.1)

^a^A total of 81 (50.2%) cases were unspecified.

### Assessment

No technical problems with the data capture system were reported over that period, and therapists noted that data entry was quick and easy, typically taking less than 2 minutes. When therapists had questions about how to enter individual interventions, support was provided to help them locate the intervention in the menu. Questions regarding interventions were infrequent and usually occurred on first attempts to enter data.

A total of 47 (57%) physical therapists used the “Clinical Notes” text box at least once to describe or list at least one intervention or to add assessment data for a daily note. Investigator review found clinical notes from 16% (428/2615) of visits’ listed interventions. Thus, 84% (n=2187) of total PT visits documented all interventions using existing system categories. The most common reason (162 visits, 6.2%) for including interventions in the text box was that the therapist exceeded the maximum allowable number (ie, 10) of strengthening exercises. In 262 (10%) visits, the physical therapist listed interventions in the text box that were available in the intervention menus, but the therapist did not choose from the menu. A total of 42 (2%) visits included interventions in the text box that were not available in the menus. In these 42 visits, there were only 5 unique interventions not available in the menus.

## Discussion

### Principal Findings

In this paper we demonstrate that it is possible to develop a structured, menu-driven data capture system to collect detailed, discrete, and quantifiable intervention data across multiple physical therapists and practice sites in patients post TKR. The system was technically reliable with no reported technical difficulties. The system’s usability is supported by the longitudinal documentation of post-TKR sessions by 83 physical therapists or physical therapist assistants across diverse PT practices. The users noted that data entry was easy and quick. One user noted that it was easier than the clinic’s own EHR.

A total of 16% (428/2615) of visits included text to describe PT interventions, but much of the text provided additional information about the patient encounter, such as objective measures of outcomes. Some of the data entries listed interventions that were available in the intervention menus that the physical therapist had not identified. One reason for this was that the system imposed a maximum of 10 interventions for the strengthening exercise category. Removing this limit will eliminate the need to document more exercises in text. Some physical therapists listed interventions that they had not found in the menus, although those interventions were available. More extensive training of the users to ensure that they are familiar with all intervention menus will further minimize the need for text entry. In only 42 (2%) of over 2600 PT visits, there were new interventions listed in the text box that were not included in the menus. If our future outcome analyses find that any of these interventions are associated with positive outcomes, they can easily be added to the menus. Overall, fewer than 2% of the thousands of visits included an intervention that was not included in the documentation system.

Prusaczyk et al [9] suggest that complex interventions may be assessed through the use of EHRs if assessors evaluate all the data found in the record including open text extraction. However, wide application of open text extraction is challenged by the absence of a common vocabulary across treatment sites and the common use of jargon. Further, our preliminary research found that physical therapists did not document all interventions, compromising the use of text extraction [8]. The use of the EHRs for clinical research or quality improvement assessments of daily practice requires that uniform data are collected using a structured format.

It is important to note that the extensive list of interventions in our data capture system was designed to provide an exhaustive list of any conceivable intervention that a physical therapist or physical therapist assistant might use with a patient post TKR. The ultimate goal of our study is to identify those interventions and treatment factors that are associated with greatest knee performance and functional outcomes. These analyses are ongoing. After those interventions and factors are identified, the data capture system can be simplified and tailored to facilitate the application of the preferred interventions. For example, the most commonly used interventions can be listed first. In addition, a future iteration of the system can incorporate real-time clinical decision support principles to encourage physical therapists to adopt interventions associated with optimal outcomes or to advance intensity and repetitions. Despite the use of our exhaustive list of interventions, the users estimated that the time for data entry was approximately 2 minutes per visit. The proposed future enhancements may further improve upon the documentation efficiency and add clinical value through recording comprehensive and specific intervention details. Last, the structure and content could be integrated with existing PT EHRs to eliminate the second log-in and assure no redundancy exists between the administrative EHR functions and consistent PT intervention documentation.

The ability to successfully capture detailed intervention data representing real-world evidence, across multiple sites and providers, enhances the potential of future CER to identify best PT practices. The current data capture system can be readily adapted for use in many populations receiving PT, where care is known to exhibit significant unexplained practice variation. Importantly, we believe that the framework we used to develop this data capture system can be applied across the health care system, with a priority on treatments for which additional comparative effectiveness evidence is needed. Our data capture system was successful for several reasons. The data capture system was intended to capture relevant and detailed clinical data. Although the intervention data could easily be mapped onto reimbursement algorithms for billing purposes, its primary focus was clinical applications. Additionally, the system was designed by individuals familiar with the health services being provided, so the information gathered was consistent with clinical practice. The process of identifying the data to be captured was iterative, involving a broader review by more potential users at each level. Finally, a dictionary of clinical interventions was generated to ensure the collection of uniform data.

### Limitations and Future Considerations

The data collection system described in this paper is a prototype; it is not integrated into any health system’s electronic medical record. Future studies will assess its effectiveness and efficiency in the real world by integrating it into existing EHRs. Although almost 100 clinicians entered data collected from over 160 patients, testing will be improved by increasing the number of therapists entering data and the number of patients whose data are recorded. Finally, some therapists did not use the available menus effectively. Improved training for clinicians and their use of the data capture system over an extended period will improve their ability to use the system effectively and efficiently.

In conclusion, we have demonstrated that a structured data capture system to collect detailed quantifiable intervention data from multiple physical therapists at multiple sites is feasible and effective. Development of the system required involvement of potential end users and broad review to ensure the collection of a uniform yet complete data set. We believe this approach can be used by multiple health care disciplines to develop data capture systems that produce real-world evidence, suitable for quality improvement processes as well as for comparative effectiveness and outcomes research. In the future, clinical registries and EHRs can adopt structured health intervention documentation taxonomies, such as we describe, to assure complete, consistent real-world evidence to accelerate the potential for learning health systems

## Appendix

Multimedia Appendix 1 List of interventions (strengthening intervention category with dose and intensity).

## Abbreviations

CER: comparative effectiveness research
EHR: electronic health record
HIPAA: Health Insurance Portability and Accountability Act
OA: osteoarthritis
PT: physical therapy
TKR: total knee replacement
